# Synthesis and Evaluation of Polyurethane as Waterproof Adhesion Layer for Steel Bridge Deck

**DOI:** 10.3390/polym16223140

**Published:** 2024-11-11

**Authors:** Yan Chen, Jianying Hu, Xiaonan Wu, Shaochan Duan, Hongyu Wang, Tao Ma

**Affiliations:** School of Transportation, Southeast University, Southeast University Road #2, Nanjing 211189, China; 220223120@seu.edu.cn (Y.C.); duanshaochan@163.com (S.D.); 220223045@seu.edu.cn (H.W.); matao@seu.edu.cn (T.M.)

**Keywords:** polyurethane WAL, steel bridge deck, crosslinking agent, acrylic co-blend, performance

## Abstract

Service life of traditional steel bridge deck pavement is significantly shortened due to the failure of waterproof adhesion. To improve the interlayer bonding performance and extend its service life, polyurethane is proposed as a waterproof adhesion layer (WAL) for a steel bridge deck. This study aims to synthesize polyurethane WAL by free radical solution polymerization under different types and dosages of crosslinking agents as well as the mixing ratio of an acrylic co-blend. Tensile properties, water resistance, glass transition temperatures, thermal stability, and adhesive strength of the polyurethane binder are evaluated. The results demonstrate that polyurethane WAL yields desirable performance by using a hydroxyl molar ratio of 1/3 and an acrylic co-blend of 65%. Specifically, the tensile strength and breaking elongation of polyurethane WAL reach the maximum values of 6.466 MPa and 268.4%, respectively. The water absorption rate of polyurethane WAL is less than 4%. Glass transition temperatures of polyurethane WAL are between −80 °C and 60 °C, respectively. Polyurethane WAL features remarkable high- and low-temperature performance and thermal stability. Finally, adhesion strength between polyurethane WAL and the steel plate reaches up to 5.21 MPa. The outcome of this study facilitates the design and application of polyurethane waterproofing adhesion layers for steel bridge decks.

## 1. Introduction

A waterproof adhesion layer (WAL) plays a crucial role in the overall performance of steel bridge deck pavement structures due to its functions as protective coating and bonding between the paving and the underlying steel plate. The deterioration and failure of waterproof adhesion materials result in a reduced service life of steel bridges. Traditional WAL materials are solvent-based asphalt waterproofing binder materials [1,2,3,4], methacrylic acid waterproofing materials [5], and epoxy binder materials [6,7,8,9]. However, these three types of traditional WAL materials exhibit limitations of complex construction processes, deficiencies in durability, and extended construction timelines. The construction procedure mandates a sequential implementation of a primer, a waterproof layer, and an adhesive layer, a methodology that is markedly prone to disruptions stemming from weather conditions [10,11,12]. This approach not only substantially increases the financial expenditures related to the project but also engenders an unwarranted consumption of energy [13,14,15]. Furthermore, it introduces an element of unpredictability with respect to the bonding performance, which may ultimately impair the structural integrity and reliability of the constructed elements. Thereafter, it is worth exploring innovative WAL materials with easy construction and enhanced performance. Polyurethane is a promising alternative material for waterproofing adhesion layers, which is synthesized by a chemical reaction between polyisocyanates and polyols with the incorporation of chain extenders and crosslinkers. It consists of flexible and elastic segments, which endow preferred deformation coordination performance with a steel bridge deck. Furthermore, polyurethane possesses urethane groups and polar groups, resulting in enhanced molecular polarity. This enables its excellent chemical adhesion with paving materials and steel decks [16]. Meanwhile, polyurethane presents characteristics of high strength, exceptional thermal stability, water resistance, and anti-corrosiveness. To improve mechanical and adhesion properties of polyurethane, researchers have proposed to dope different kinds of additives, such as pigments [17], nanoparticles [18,19,20], and chain extenders [21,22,23,24]. Polyurethanes are versatile block copolymers whose material properties are intricately linked to their molecular structure [25,26]. One effective strategy for enhancing adhesive properties of polyurethane is the replacement of polyols with partial chain extenders. Chain extenders containing hydroxyl (-OH) functional groups exhibit high reactivity with isocyanate groups, which thereafter affect adhesion performance by altering the relationship between hard and soft segments of polyurethane. It is stated that both the composition and chemical structure of the chain extenders yield a great contribution to adhesion performance of polyurethanes [27,28,29]. Tan [21] reported that chain extenders affect cohesive strength of polyurethane by modulating the degree of microphase separation between hard and soft segments. Wang [22] systematically analyzed effects of crosslinker and chain extender dosages on the bi-directional shape memory deformation and mechanical properties of crack sealants. By investigating the effect of the hydroxyl value on the adhesive, P. N. Moghadam [30] found that as the hydroxyl value of the polyol increased, a linear increase in the lap shear strength of the adhesive joint was observed. Hence, adhesion performance of polyurethane could be modulated by designing its chemical constituents.

This study aims to synthesize a polyurethane binder through free radical polymerization, which is proposed as a waterproofing adhesion layer (WAL) for steel bridge deck pavement. To formulate polyurethane WAL, two types of crosslinking agents are utilized at different dosages, including trimethylolpropane (TMP) and hydroxyethyl acrylate (HEA). Additionally, an acrylic co-blend is used as a diluent for the preparation of a polyurethane prepolymer. Moreover, performance of polyurethane WAL is systematically evaluated, including tensile properties, water resistance, adhesion strength, and thermal stability. Finally, the formulation of polyurethane WAL material is optimized for its application in a steel bridge deck.

## 2. Materials and Methods

### 2.1. Raw Materials

Polypropylene glycol (PPG, Mn = 1000 g/mol, 105.62 mgKOH/g), 4,4′-methylene bis (phenyl isocyanate) (MDI, Mn = 250.25 g/mol), trimethylolpropane (TMP, Mn = 134.17 g/mol), hydroxyethyl acrylate (HEA Mn = 116.12 g/mol), methyl methacrylate (MMA, Mn = 128.169 g/mol), and butyl acrylate (BA, Mn = 100.12 g/mol) were provided by Sinopharm chemical reagent Co., Ltd. (Shanghai, China). Benzoyl peroxide (BPO) and *N*,*N*-dimethylacetamide (DMA) were purchased from Shanghai Macklin Biochemical Technology Co., Ltd. (Shanghai, China); dibutyl acetate, dibutyl amine, and dibutyltin dilaurate (DBTDL) were purchased from Shanghai Aladdin Biochemicals. The dosage of each material is shown in Table 1. Table 2 shows chemical data for MMA and BA.

### 2.2. Synthesis of Polyurethane Waterproofing Adhesion Layer (WAL)

**Figure 1:**
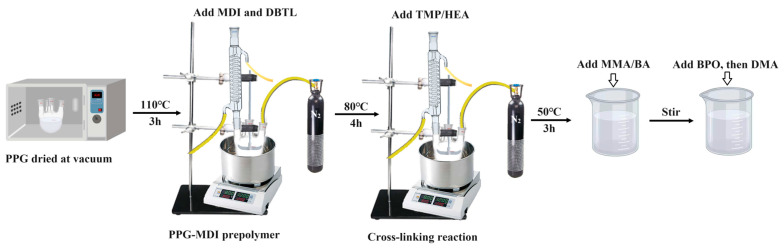
Preparation process of polyurethane WAL.

**Figure 2:**
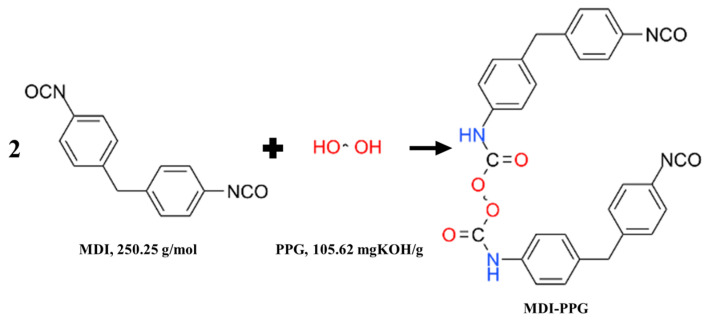
Synthesis process of PPG-MDI.

**Figure 3:**
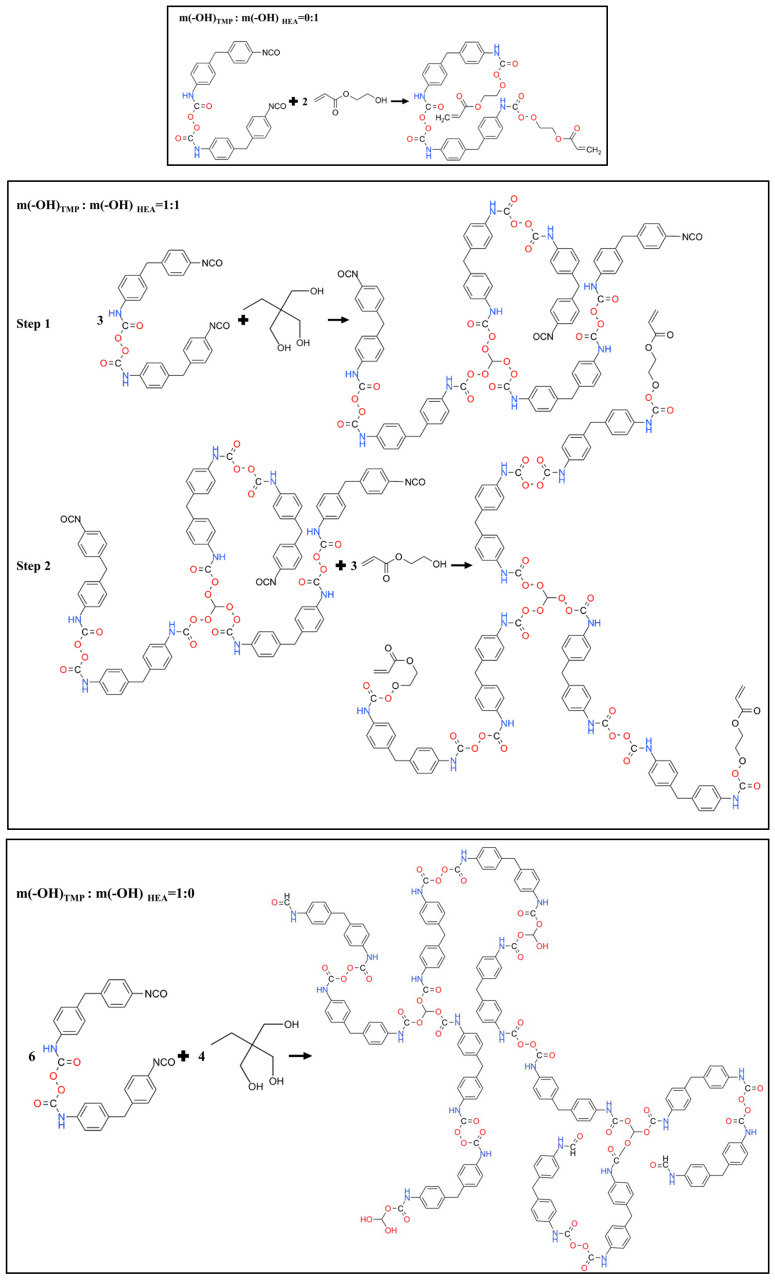
Synthesis process of polyurethane prepolymer.

Polyurethane WAL was synthesized through the free radical solution polymerization method, as depicted in Figure 1. Initially, a certain quantity of PPG was dehydrated under vacuum at 110 °C for 3 h and subsequently preheated to 80 °C. Then, MDI at the designed dosage was gradually added into PPG. Meanwhile, the catalyst (DBTDL) at a concentration of 0.03% was doped. The mixture was stirred at 80 °C for 4 h under a nitrogen atmosphere. The endpoint of this reaction was determined using the butyl acetate-dibutylamine back-titration method. The relevant chemical reaction of the process is shown in Figure 2. Next, when the reaction temperature dropped to 50 °C, a crosslinking agent was incorporated into the mixture to form a polyurethane prepolymer with a crosslinked network structure. It was further stirred at 50 °C under nitrogen for 3 h. The corresponding chemical reactions during this stage are presented in Figure 3. Finally, the polyurethane prepolymer at a specific dosage was blended with the diluent at room temperature. Curing initiators and accelerators were also added to facilitate the curing of the redox system, using 0.8% DMA and 2% BPO. Polyurethane WAL was thereafter fabricated.

During the synthesis of polyurethane WAL, two types of crosslinking agents (TMP and HEA) were employed. The hydroxyl molar ratios of TMP to HEA were designed at 0:1, 1:3, 1:1, 3:1, and 1:0, which are designated as PU1, PU2, PU3, PU4, and PU5, respectively. Tensile properties, thermal stability, water resistance, and adhesion to the steel plate were comparatively analyzed across five formulations. Crosslinking agent formulation was then optimized based on the performance evaluation of polyurethane WAL.

It is widely recognized that methyl methacrylate (MMA) and butyl acrylate (BA) are individually employed as soft and hard monomers for fabricating acrylic rubber with specific flexibility and tensile strength [31]. Wu et al. [32] proposed the addition of 30% to 70% MMA as the diluent for polyurethane-based chemicals. In this study, both MMA and BA were incorporated to effectively control curing time of polyurethane adhesives, ensuring both fluidity and ease of construction. A previous study employed the total mass of MMA and BA by 65% of the total mass of the polyurethane prepolymer [33]. To delve deeper into the influence of MMA and BA on the characteristics of polyurethane WAL within acrylic blends, a series of five experimental schemes were meticulously crafted. These schemes were designed with varying proportions of MMA, which constituted 100%, 75%, 50%, 25%, and 0% of the total acrylic blend, respectively.

### 2.3. Characterizations of Polyurethane WAL

#### 2.3.1. Tensile Property

**Figure 4:**
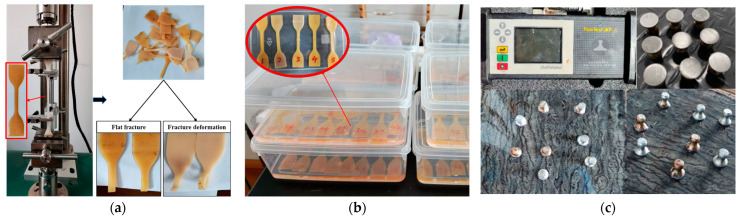
(**a**) Tensile tests, (**b**) water immersion tests, and (**c**) adhesion pull-off tests.

A tension test was performed to evaluate tensile properties of polyurethane WAL in accordance with the standard of ASTM D638 [34]. It was implemented with a universal testing machine (HF-9006, Jiangsu Ligao Test Equipment Co., Ltd., Suzhou, China) with a tension rate of 50 mm/min at a temperature of 23 °C. The dumbbell-shaped specimens were fabricated, as depicted in Figure 4a. For each polyurethane WAL, at least five samples were tested. Tensile strength of the sample is calculated as Equation (1):

(1) $$T_{L}=\frac{P}{BD}$$

where $T_{L}$ (MPa) is tensile strength; P (N) is the maximum tensile force; and B and *L* (mm) are the width and thickness of the specimen, respectively.

The elongation at break is calculated as in Equation (2):

(2) $$E=\frac{L_{1}-L_{0}}{L_{0}}\times 100$$

where E (%) is the elongation at break; $L_{0}$ (mm) is for the specimen start line spacing; and L (mm) is for the specimen fracture line spacing.

The retention rate of the tensile property is calculated according to Equation (3):

(3) $$R_{t}=\frac{T_{1}}{T}\times 100\%$$

where $R_{t}$ (%) is the retention rate of tensile properties; T is the average value of tensile strength before sample treatment; and $T_{1}$ is the average value of tensile strength after sample treatment.

#### 2.3.2. Water Resistance

Polyurethane is susceptible to hydrolysis under water, which impairs adhesion between polyurethane WAL and a steel plate. In this study, hydrolysis of polyurethane WAL is evaluated by water resistance tests in accordance with the standard of GB/T 1034-2008 [35]. These tests involved monitoring the change in mass and tensile retention rate of the samples that were submerged in deionized water at a controlled temperature of 23 °C (±1 °C) for varying durations (0, 1, 2, 3, 4, 5, 6, and 7 days), as illustrated in Figure 4b. Water absorption (*ω*) was calculated according to Equation (4):

(4) $$\omega =\frac{m_{i}-m_{0}}{m_{0}}\times 100\%$$

where $m_{0}$ and $m_{i}$ are the mass of the specimen before and after water immersion at i days.

#### 2.3.3. High- and Low-Temperature Performance

Steel plates used in bridge decks present large thermal conductivity, which yields a high surface temperature of bridge deck pavement. It has been reported that surface temperature of steel bridge deck pavement is typically 5 °C higher than surface temperature of the surrounding road during the summer. Additionally, due to the rapid heat loss by steel plates, bridge deck pavement is more prone to ice formation in winter. To apply a polyurethane binder in a steel bridge deck, it is necessary to evaluate high- and low-temperature performances of polyurethane WAL. In this study, high-temperature performance of polyurethane WAL was assessed by measuring tensile strength of the sample subjected to heating at 60 °C (±0.5 °C) for varying durations up to 7 days. Low-temperature performance of polyurethane WAL was evaluated by measuring the tensile strength retention of the sample subjected to freezing in a refrigerator at 0 °C (±0.5 °C) for different periods up to 7 days.

#### 2.3.4. Thermal Analysis

Glass transition temperatures of polyurethane WAL were investigated by implementing differential scanning calorimetry (DSC) tests. DSC tests were carried out under a nitrogen atmosphere with a gas flow rate of 20 mL/min using DSC Q2000 (New Castle, DE, USA). Temperature during the DSC test ranged from −90 °C to 150 °C. The samples with weights of 5–10 mg were firstly heated to 150 °C for 5 min and then cooled down to −90 °C for another 5 min, which were then heated back up to 150 °C. The rate of temperature change in this program was set as 10 °C/min. All data were obtained from the second scan to erase the previous thermal history [33]. Glass transition temperatures of the sample were confirmed by analyzing heat flow curves taken during the second heating cycle.

A thermogravimetric analysis (TG) was adopted to examine thermal stability of polyurethane WAL. It measures the weight of the sample varying with increasing temperature under controlled conditions. The analysis in this study was performed on the sample with a weight of 3 mg using a NETZSCH TG 209F3 analyzer (NETZSCH-Gerätebau GmbH, Selb, Germany). Testing temperature was raised from 30 °C to 620 °C at a rate of 20 K/min.

#### 2.3.5. Adhesion Between Polyurethane WAL and Steel Plate

In accordance with the specification in GB/T 5210–2006 [36], adhesion between polyurethane WAL and a steel plate was examined. The polyurethane binder was firstly sprayed onto a steel plate with dimensions of 1.2 cm × 50 cm × 50 cm, which was cured and dried completely. After that, the pull-type adhesion test was conducted on the sample to measure bonding strength between polyurethane WAL and the steel plate, as illustrated in Figure 4c. This test was performed at room temperature (23 °C ± 1 °C). Following the recommendations in the JTG/T3364-02-2019 [37], the application rate of polyurethane WAL was set between 0.1 and 0.6 kg/m^2^.

## 3. Results and Discussion

### 3.1. Tensile Property of Polyurethane WAL

**Figure 5:**
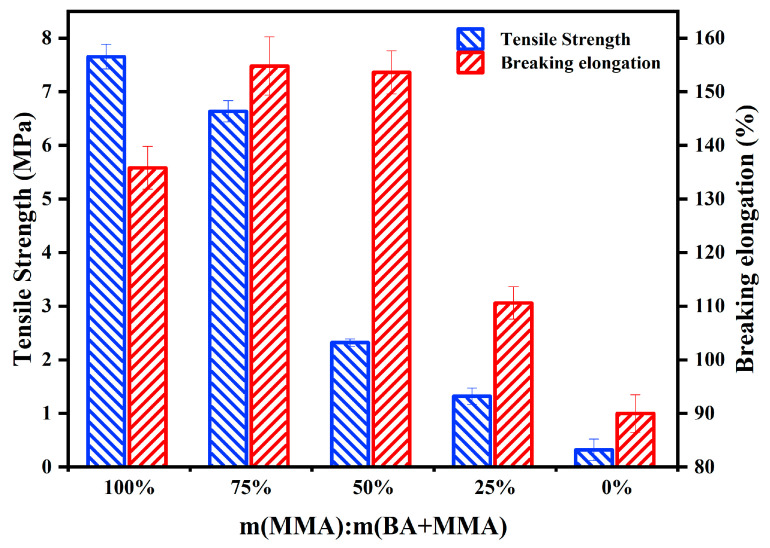
Effect of diluent on tensile properties of polyurethane WAL.

In this study, the blended solution of MMA/BA with different proportions was selected as the diluent for the synthesis of polyurethane WAL. Figure 5 illustrates the effect of the diluent with different compositions on tensile properties of polyurethane WAL. As depicted in Figure 5, an increase in tensile strength of polyurethane WAL was observed with increasing MMA/BA content, which agrees with observations in previous studies [38]. Tensile strength of polyurethane WAL reaches up to the maximum value of 7.8 MPa when the MMA dosage is 65% while its tensile strength is less than 3 MPa when the MMA and BA dosages are half of each. This is attributed to a higher surface tension of BA, which leads to a better dilution effect and thereafter lowers the tensile strength of polyurethane WAL. Furthermore, as the dosage of BA rises, the elongation break of polyurethane WAL rises firstly and then drops. This is associated with the molecular structure of the diluent. Due to the linear structure and larger molecular weight as well as long chain length of BA, BA as a soft monomer can endow a polymer with good flexibility. The MMA as a hard monomer could endow a polymer with good hardness and tensile stress. However, the use of a large amount of BA leads to reduced tensile strength of a polymer while the doping of a large amount of MMA results in lower failure strain of a polymer [31]. When the mass ratio of MMA is 0.75, the elongation at break reaches the maximum value of 156%. To achieve the preferred tensile property of polyurethane WAL, it is recommended to use a diluent mixture consisting of 49% MMA and 16% BA in an acrylic blend.

**Figure 6:**
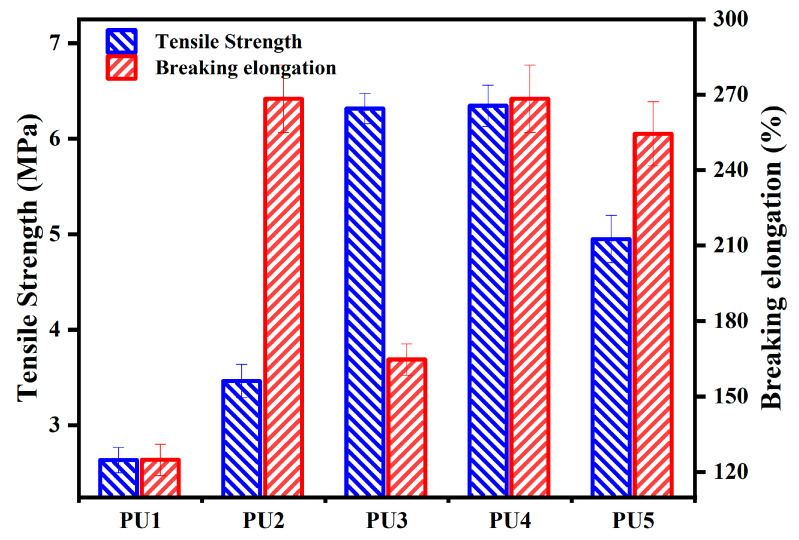
Effect of crosslinker type and dosage on tensile properties of polyurethane WAL.

Numerous studies demonstrated that although content of chemical crosslinking was low, its molecular structure imposed a significant impact on the performance of a polymer [39,40,41]. For MDI-based polyurethane, TMP used as a crosslinker affects both tensile strength and elongation at break [42,43,44]. The effect of the crosslinker type and dosage on the tensile property of polyurethane WAL is presented in Figure 6. As depicted in Figure 6, tensile strength of the specimens increases with the addition of crosslinker TMP. When the hydroxyl molar ratio of TMP: HEA is 1:1 (PU3), tensile strength of polyurethane WAL is significantly enhanced. When the hydroxyl molar ratio is 3:1 (PU4), tensile strength reaches the maximum. Moreover, a marginal decrease was observed in tensile strength of polyurethane WAL when TMP was used exclusively as a crosslinker. For this sample, the cross-section at failure is not as flat as that in PU1, indicating increased toughness of PU5 [45,46]. Additionally, the breaking elongation of polyurethane WAL shows an increasing trend with the increase in the hydroxyl molar ratio of TMP/HEA. It can be found that the elongation at break of PU2 is significantly larger than that for PU1. Moreover, breaking elongation for the samples is in the order of PU2 > PU4 > PU5 > PU3 > PU1, while tensile strength of the samples is in the order of PU4 > PU3 > PU5 > PU2 > PU1.

Taking into account both tensile strength and elongation at break, it is evident that when the hydroxyl molar ratio of TMP to HEA is 3:1 (PU4), tensile performance is excellent. This is due to the presence of three hydroxyl groups in the structure of TMP, which facilitates the reaction with isocyanate to form polyurethane. Moreover, the branched-chain structure in TMP enhances the spatial mesh crosslinking structure of the polyurethane binder and thereafter improves its tensile strength. However, the solid state of TMP at room temperature also poses challenges to workability of the sample during the preparation process. In addition, the presence of C=C double bonds in the molecular structure of HEA, with greater bond energy, could contribute to improving the strength of polyurethane WAL.

### 3.2. Water Resistance

**Figure 7:**
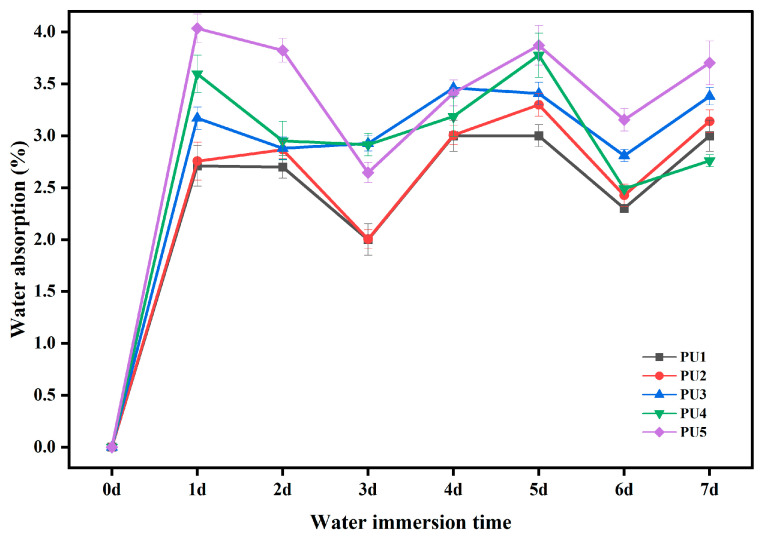
Water absorption of polyurethane WAL under different periods of water immersion.

Figure 7 illustrates water absorption rates of the polyurethane waterproofing adhesive (WAL) after various durations of water immersion. It is evident that water absorption rates of samples remained at less than 4.0% after immersion, indicating that polyurethane WAL possesses excellent water resistance. This finding aligns with results in the existing literature [45]. With increasing content of the crosslinking agent of TMP, the water absorption rate of polyurethane WAL gradually increased. This is attributed to three hydrophilic hydroxyl groups in TMP, which enhance the binding of water molecules to the surface of polyurethane WAL and thereby impact its performance. To mitigate water absorption, some studies have used HEA as a capping end to prepare polyurethane prepolymers, which decreases hydrophilicity and improves water resistance of polyurethane adhesives [47]. The double bond structure at the side end of hydroxyethyl acrylate increases the stability of the material, which reduces the combination with water molecules to a certain extent and decreases the water absorption rate [48,49]. However, an increase in the dosage of acrylate monomers affects the adhesion properties of polyurethane WAL [50]. Furthermore, it can be seen that during the initial three days of water immersion, the water absorption of polyurethane WAL is decreased. This is due to the free isocyanate in the polyurethane binder reacting with water to generate carbon dioxide, which results in a lower mass and water absorption.

**Figure 8:**
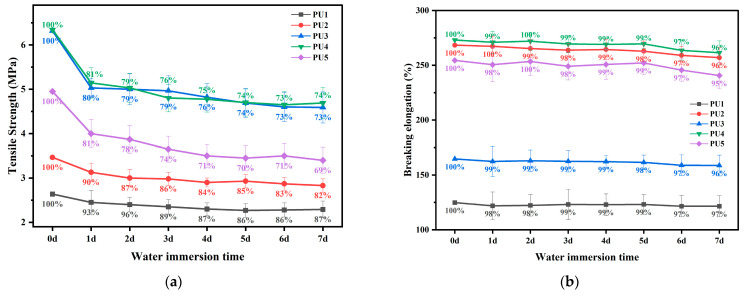
Tensile strength (**a**) and breaking elongation (**b**) of polyurethane WAL under different periods of water immersion.

Figure 8 exhibits the effect of water immersion on tensile properties of polyurethane WAL. With the prolongation of water immersion, the elongation at break and tensile strength of polyurethane tend to decrease. More importantly, with the increase in TMP content, the effect of water immersion on tensile properties of polyurethane waterproof adhesive materials increases [51,52]. When the ratio of TMP: HEA is 0: 1, the retention rate of tensile strength and elongation at break after 7d of water immersion for polyurethane WAL are 87% and 97%, respectively. When the ratio of TMP to HEA is 1:0, these retention rates are 69% and 95%, respectively. This could be explained in that the samples soften after water immersion and present decreased hardness and brittleness. Thus, the sample presents a more rapid decrease in tensile strength than elongation at break. Additionally, water molecules infiltrate the surface of the samples after 3 days of water immersion, causing a partial dissolution and decrease in the measured mass of the samples. With further water immersion, the sample reaches the saturation of water absorption. The amount of free isocyanate groups that can react with water gradually decreases, which leads to mass change in the spline after water absorption. Therefore, to lower the water absorption of polyurethane WAL, the molar ratio of TMP to HEA needs to be controlled to be less than 3. In this way, a dense network crosslinking structure can be formed in the polyurethane binder to ensure its tensile properties and water resistance [45,46].

### 3.3. High- and Low-Temperature Performance of Polyurethane WAL

**Figure 9:**
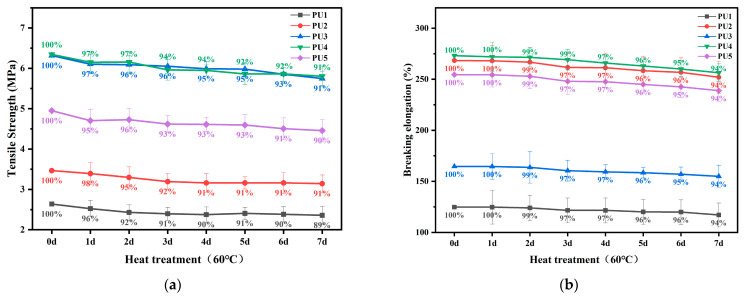
Tensile strength (**a**) and breaking elongation (**b**) of polyurethane WAL after thermal treatment at 60 °C.

Figure 9 presents the tensile strength and breaking elongation of polyurethane WAL after thermal treatment at 60 °C. It is evident that with an increase in the duration of thermal treatment, both the tensile strength and breaking elongation of polyurethane WAL drop. Using Equation (3), the retention rate of tensile strength of the samples was calculated. It is revealed that after 7 days of thermal treatment, the retention rate of tensile strength of the samples remained above 90%. Meanwhile, the retention rate of breaking elongation is greater than 94%. This implies that polyurethane WAL exhibits desirable high-temperature performance during summer. Among various types of polyurethane WALs, PU4 demonstrates a superior retention of tensile properties after heat treatment. This indicates that when the hydroxyl molar ratio of TMP to HEA is designed as 3:1, a crosslinked mesh structure is formed in polyurethane, which significantly improves its high-temperature performance. This observation is further supported by the analysis of DSC and TG curves [33,45].

**Figure 10:**
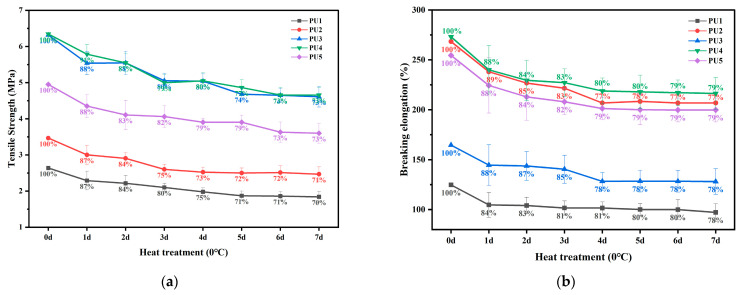
Tensile strength (**a**) and breaking elongation (**b**) of polyurethane WAL after 0 °C treatment.

Figure 10 shows the tensile strength and breaking elongation of polyurethane WAL after freezing treatment. It is noted that the decay of tensile properties gradually becomes slower with the duration of freezing treatment. As shown in Figure 10a, after one day of freezing treatment, retention rates of tensile strength for PU1, PU2, PU3, PU4, and PU5 are 87%, 87%, 88%, 91%, and 88%, respectively. The retention rate of tensile strength for them is only 70–73% after freezing treatment at 0 °C for 7 days. A low temperature yields a larger impact on the tensile property of polyurethane WAL compared with a high temperature. Additionally, it can be seen from Figure 10b that the effect of low-temperature treatment on the breaking elongation of polyurethane is more significant. This is due to the reduced flexibility of polyurethane at low temperatures.

By comparing Figure 9 and Figure 10, it is observed that a low temperature imposes more impact on tensile properties of the polyurethane binder than a high temperature. This is closely related to the molecular structure of the polyurethane binder. According to previous studies, PU4 forms a network crosslinking structure, which accounts for its exceptional resistance to weathering. In contrast, PU1 lacks a network crosslinking structure due to the presence of a tri-hydroxyl group and exhibits deteriorated tensile properties. It is also susceptible to temperature, showing lower tensile properties after thermal treatment.

### 3.4. Glass Transition Temperatures of Polyurethane WAL

**Figure 11:**
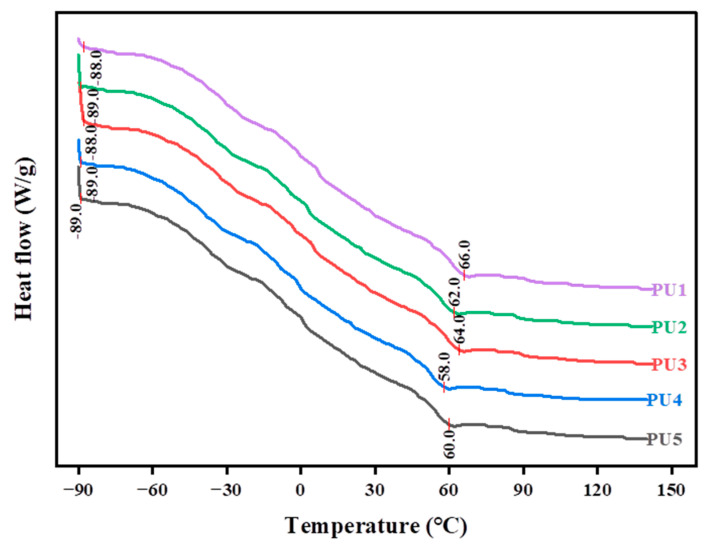
DSC diagrams of polyurethane WAL with different types and dosages of crosslinker.

Figure 11 presents DSC curves of polyurethane WAL with different types and dosages of a crosslinker. It is observed that the negative glass transition temperature of the specimens is lower than −80 °C. This is attributed to characteristics of polyol-based soft segments in a polyurethane binder [53]. The positive glass transition temperature is greater than 60 °C. Duan et al. [33] observed the positive glass transition temperature above 50 °C by analyzing the DSC curve of polyurethane materials. Xing et al. [54] found that the endotherm peak of polyurethanes is between 60 °C and 80 °C. Additionally, the positive glass transition temperature is the highest when HEA is used entirely as the crosslinking agent (PU1), which reaches up to 66 °C. The lowest positive glass transition temperature is 58 °C for PU4. When TMP (PU5) is used as the crosslinker, the negative glass transition temperature is −89 °C, and polyurethane WAL has excellent low-temperature flexibility. However, the glass transition temperature of polyurethane WAL is not only dependent on chemical crosslinking, but also on the type of soft segment, the relative molecular mass of the soft segment, the type of hard segment, the mass fraction of hard and soft segments, and the synthesis method.

### 3.5. Thermal Stability

**Figure 12:**
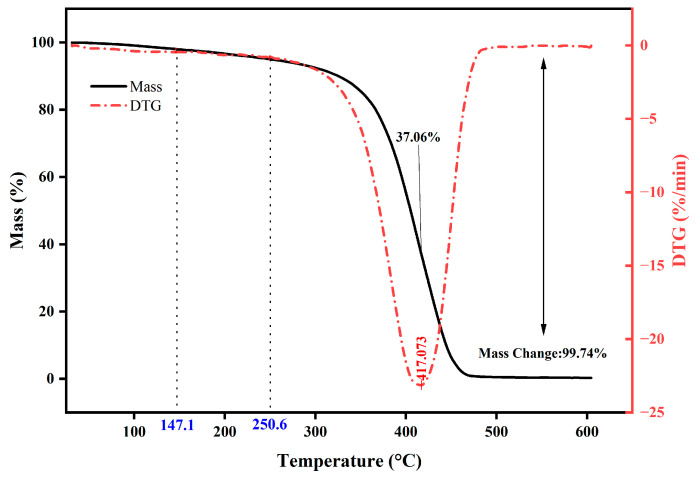
TG and DTG curves.

Figure 12 illustrates thermogravimetric (TG) curves and thermogravimetric differential (DTG) curves for polyurethane WAL with superior performance (PU4). Weight loss of the sample is found to be 2 % and 5 % at decomposition temperatures of 147.1 °C and 250.6 °C, respectively. These temperatures are significantly higher than temperatures of bridge deck pavement in service, indicating its thermal stability [32,55]. It is also observed in DTG curves that the maximum decomposition rate occurs at 417 °C, which corresponds to the breaking of the hydrocarbon main chain and a 37% mass change in the sample. In summary, the polyurethane waterproofing binder prepared in this study possesses superior thermal stability, meeting the criteria for use in bridge deck pavement.

### 3.6. Adhesion Between Polyurethane WAL and Steel Plate

**Figure 13:**
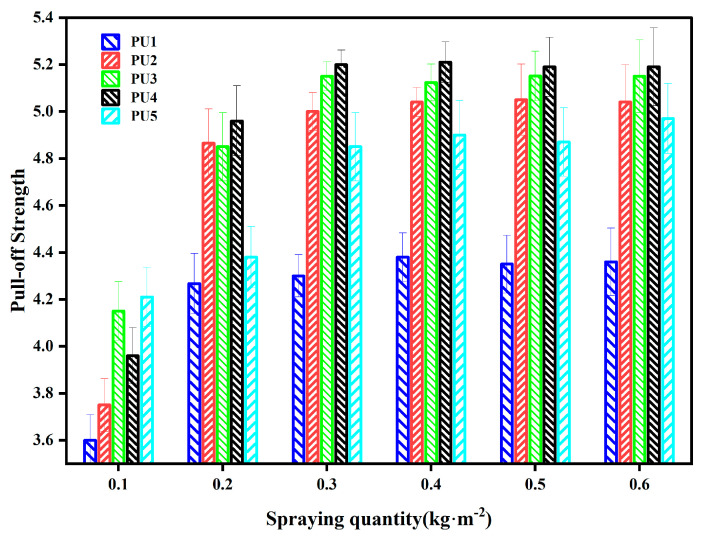
Adhesion pull-off strength of polyurethane WAL under different spraying rates.

Figure 13 presents the pull-off strength of polyurethane WAL at various application rates. The adhesion pull-off strength for all samples exceeded 3.0 MPa, which is close to a result in the literature [55]. The strong hydrogen bonds are formed by the hydroxyl and urethane groups in WAL [32], which further improve the bond strength of polyurethane WAL to a steel plate. As the spraying quantity of polyurethane WAL rises, pull-off strength initially rises and then remains constant. This trend may be attributed to the presence of air bubbles or defects in the thicker polymer layer, which can impair tensile strength of the sample at the interface and bring pull-off failure [56]. When the spraying rate of polyurethane WAL is large, the adhesion of polyurethane WAL to the steel plate is strong. Therefore, it is suggested to apply polyurethane WAL at a rate between 0.3 kg/m^2^ and 0.4 kg/m^2^ to ensure adhesion.

## 4. Conclusions

This study was dedicated to the development of polyurethane as waterproofing adhesives (WALs) in steel bridge deck paving. The synthesis process entailed the reaction of methylene diphenyl diisocyanate (MDI) with polypropylene glycol (PPG). Within this synthesis, an acrylic acid copolymer served as a diluent, and two crosslinking agents (TMP and HEA) were employed in varying ratios. Tensile strength, water resistance, glass transition temperatures, thermal stability, high- and low-temperature performances, and adhesion strength of polyurethane WAL were evaluated. The main conclusions are as follows:

- When the MMA to BA ratio is 3:1, the elongation at break of polyurethane WAL reaches the maximum value of 156%, while tensile strength is 6 MPa. To attain the desired tensile properties of polyurethane WAL, it is recommended to utilize a diluent of a co-mingled solution comprising a 49% MMA and 16% BA acrylic blend.
- Two distinct reagents were employed as crosslinking agents for polyurethane WAL. It revealed that when the hydroxyl molar ratio of TMP: HEA is 3:1, tensile properties of polyurethane WAL were desirable with a breaking elongation of 273% and tensile strength of 6.466 MPa.
- Polyurethane WAL demonstrated exceptional water resistance as its water absorption rate after 7 days of immersion at 23 °C was less than 4%, and its tensile properties exceeded 70%.
- Polyurethane WAL exhibited remarkable tensile properties even after exposure to extreme temperatures. After heat treatment at 60 °C, it retained over 90% of its tensile properties, and after treatment at 0 °C, it retained over 70% of its tensile properties.
- Based on the DSC analysis, the negative glass transition temperature of polyurethane WAL was lower than −80 °C and its positive glass transition temperature was larger than 60 °C. TG revealed that the thermal decomposition temperature of polyurethane WAL was about 250 °C, implying its superior thermal stability.
- Polyurethane WAL presented remarkable adhesion to steel plates, which possess adhesion strength of up to 4 MPa at an application amount of 0.3–0.6 kg/m^2^. Thereafter, polyurethane binders could be used as waterproof bonding layer material for steel bridge decks.

## Figures and Tables

**Table 1:** Dosage of raw Materials.

Description of Samples	MDI		PPG		TMP		HEA		MMA	BA
	mol	Mass/g	mol	Mass/g	mol	Mass/g	mol	Mass/g	Mass/g	Mass/g
PU1	0.2	107	0.1	51	0.067	8.9	0	0	81.4	27.1
PU2	0.2	107	0.1	51	0.05	6.7	0.05	5.8	83.1	27.7
PU3	0.2	107	0.1	51	0.033	4.5	0.1	11.6	84.9	28.3
PU4	0.2	107	0.1	51	0.017	2.2	0.15	17.4	86.6	28.9
PU5	0.2	107	0.1	51	0	0	0.2	23.2	88.3	29.4

**Table 2:** Properties of MMA and BA.

Reagent	Molecular Mass	Isotonic Volume (90.2 K)	Surface Tension (dyne/cm)	Number of Rotatable Bonds
Methyl methacrylate (MMA)	100.116	240.9	23.5	2
Butyl acrylate (BA)	128.169	324.3	26.7	5

## Data Availability

The original contributions presented in the study are included in the article, further inquiries can be directed to the corresponding author.
